# The relationship between nonsuicidal self-injury, suicidal behaviour and life events among adolescents

**DOI:** 10.1192/j.eurpsy.2022.380

**Published:** 2022-09-01

**Authors:** L.O. Horváth, D. Győri, D. Komáromy, G. Mészáros, D. Szentivanyi, J. Balazs

**Affiliations:** 1 Eötvös Loránd University, Institute Of Psychology, Budapest, Hungary; 2 Pedagogical Assistance Services, -, Budapest, Hungary; 3 Eötvös Loránd University, Doctoral School Of Psychology, Budapest, Hungary; 4 Vrije Universiteit Amsterdam, Faculty Of Behavioural And Movement Sciences, Amsterdam, Netherlands; 5 Universiteit van Amsterdam, Faculty Of Social And Behavioral Sciences, Amsterdam, Netherlands; 6 Semmelweis University, Faculty Of Medicine, Department Of Psychiatry And Psychotherapy, Budapest, Hungary; 7 Semmelweis University, Mental Health Sciences School Of Ph.d, Budapest, Hungary; 8 Bjørknes University College, Oslo, Norway

**Keywords:** nonsuicidal self-injury, adolescence, life events, suicidal behaviour

## Abstract

**Introduction:**

Nonsuicidal self-injury (NSSI) is highly prevalent in clinical and non-clinical adolescent populations. Non-clinical studies focus on high school students thus vocational school students are underrepresented in research and prevention programs, despite being exposed to higher levels of stressful life events, a factor associated with NSSI and suicide.

**Objectives:**

This study aimed to explore NSSI, suicidal behavior and life events among adolescents in clinical and non-clinical, i.e. both high school and vocational school settings.

**Methods:**

A clinical (n=202) and non-clinical (n=161) sample of 13-18-year-old adolescents were assessed with the Mini International Neuropsychiatric Interview Kid, the Deliberate Self-Harm Inventory, and the Life Events List. Data were analyzed with R version 3.6.1., using Wilcoxon tests and negative binomial regression models.

**Results:**

The prevalence of suicidal behavior (W=7.306, p<.001), NSSI (W=9.652, p<.001), and life events (W=10.410 p<.001) were significantly higher in the clinical than in the non-clinical group. The relationship between NSSI and suicidal behaviour was significantly stronger in the clinical group (95% CI: [.56,.72]) than in the nonclinical group (95% CI: [.24,.52]). The interaction between NSSI and life events (Χ^2^(1)=10.49, p<.01) was associated with suicidal behavior. Interpersonal events were associated with both suicidal behavior and had a moderating effect on the NSSI–suicidal behavior relationship.

**Conclusions:**

NSSI is highly prevalent and is strongly associated with suicidal behavior in clinical and non-clinical adolescent populations. Our result call attention to the necessity of including adolescents from various educational settings in NSSI research and prevention projects during which life events, especially interpersonal events, might require special attention.

**Disclosure:**

No significant relationships.

